# Individual reflections

**Published:** 2008

**Authors:** Sreedhar K. Krishna

**Affiliations:** Faculty of Medicine, Imperial College London



